# Elevating MHC I expression on tumor cells by nanovesicles loading tyrosine kinase inhibitors can improve the efficacy of cancer vaccines

**DOI:** 10.3389/fimmu.2025.1653533

**Published:** 2025-09-11

**Authors:** Huimin Xie, Lin Ma, Xiaoli He, Songsong Zhao, Jin Wang, Ao Zhu, Changming Liu, Olga Piskareva, Chao Deng, Fenghua Meng, Mi Liu

**Affiliations:** ^1^ College of Pharmaceutical Sciences, Soochow University, Suzhou, Jiangsu, China; ^2^ Kunshan Hospital of Traditional Chinese Medicine, Kunshan, Jiangsu, China; ^3^ Institute of Minimally Invasive Thoracic Cancer Therapy and Translational Research, Soochow University, Suzhou, Jiangsu, China; ^4^ Jiangsu Province Engineering Research Center of Precision Diagnostics and Therapeutics Development, Soochow University, Suzhou, China; ^5^ Suzhou Ersheng Biopharmaceutical Co., Ltd, Suzhou, China; ^6^ Biomedical Polymers Laboratory, College of Chemistry Chemical Engineering and Materials Science Soochow University, Suzhou, China; ^7^ Wuxi Boston Biopharmaceutical Co., Ltd., Wuxi, China; ^8^ Department of Anatomy and Regenerative Medicine, Tissue Engineering Research Group, Royal College of Surgeons in Ireland University of Medicine and Health Sciences, Dublin, Ireland

**Keywords:** MHC I upregulation, redox-responsive nanovesicles, tyrosine kinase inhibitors, sunitinib, sorafenib

## Abstract

**Introduction:**

Cancer vaccines work through activating tumor-specific T cells, which can specifically attack cancer cells by recognizing antigens binding with Major-Histocompatibility-Complex I (MHC I) molecules. The downregulation or loss of MHC I expression on tumor cells can affect the efficacy of cancer vaccines.

**Methods:**

Herein, to increase the MHC I expression on tumor cells, a nanovesicle-based strategy was developed to improve the efficacy of  cancer vaccines. Several clinically applied medicines, such as tyrosine kinase inhibitors (TKIs), were screened for their capacity to upregulate MHC I.

**Results:**

Two TKIs, Sunitinib and Sorafenib, were found to be very effective in elevating MHC I expression, and they were encapsulated into redox-responsive nanovesicles respectively (SUN-KD10 or SOR-KD10), which demonstrated favourable tumor-targeting capabilities in the tumor microenvironment. Sunitinib or Sorafenib activates the IFNγ/STAT1 pathway, which improve the expression of MHC I. When combined with whole-tumor-antigen-loaded nanovaccines, these nanovesicle formulations elicited a synergistic antitumor effect in both breast cancer and melanoma mouse models. The tumors in the tumor-bearing mice treated with combined strategy grew more slowly and the survival times of such mice are significantly prolonged.

**Discussion:**

The studies demonstrated that more tumor-specific T cells were activated in the combined strategy treated mice, suggesting improved immune-mediated tumor clearance. This combinatorial approach provides a promising strategy to overcome immune evasion and to enhance the therapeutic outcomes of cancer vaccine-based immunotherapy by using clinical-applied medicines with cancer vaccines.

## Introduction

Recent progress in cancer treatment, particularly with the advance of targeted therapies and immunotherapy, has highlighted their transformative potential. Despite advances, cancer remains a leading cause of death worldwide, driven by challenges such as tumor diversity, the intricate tumor microenvironment, and individual patient variations. While immunotherapy has notably improved outcomes for patients with blood cancer through immune checkpoint inhibitors, its effectiveness in treating solid tumors is still limited. One of the primary obstacles to successful immunotherapy in solid tumors is immune evasion, often associated with reduced or absent expression of Major Histocompatibility Complex I (MHC I) genes (1–10). Cancer vaccines represent a promising approach in immunotherapy. These vaccines work by stimulating tumor-antigen-specific T cells capable of identifying and attacking cancer cells. Specifically, CD8^+^ T cells recognize tumor antigens presented by MHC I molecules, while CD4^+^ T cells identify antigens shown by Major Histocompatibility Complex II (MHC II) molecules. While MHC I molecules are broadly expressed on most cell types except red blood cells, whereas MHC II is predominantly found on antigen-presenting cells (APCs). This means that, in theory, all tumor cells have the potential to express MHC I molecules, and the presence or level of these molecules on tumor cell surfaces can significantly influence the effectiveness of cancer vaccines (4, 5, 8–10).

The downregulation or deletion of MHC I molecules on tumor cells is a critical factor influencing poor prognosis and resistance to cancer immunotherapy because T cells can not recognize tumor cells with low expression of MHC I molecules (4, 11–14). MHC I molecules play a vital role in the immune system’s ability to kill cancer cells. Their reduced expression on the surface of tumor cells allows these cells to evade detection by cytotoxic T lymphocytes (CTLs), which are essential for effective antitumor immune responses. Therefore, strategies aimed at increasing MHC I expression on solid tumor cells are crucial for overcoming immune escape mechanisms and improving the effectiveness of immunotherapy. MHC I expression on tumor cells is highly variable and can be influenced by several factors, such as Th1-secreted (T helper cells) cytokines (2) and TKIs (15) which have been shown to enhance MHC I expression by activating the IFN-γ/STAT1 signaling pathway (15–17). Herein, various drugs, including sorafenib, sunitinib, lenvatinib, ibrutinib, 5-azacytidine (5-Aza), decitabine (DAC), chloroquine (CQ), or doxorubicin (Dox), were employed to increase MHC I expression, furthermore optimized drugs were investigated in combining with cancer vaccines (7–10, 15, 18–22). Considering many chemicals potentially have severe side effects and toxicities in the clinic, the drugs we tested are all FDA-approved drugs and used in the clinic for many years with well-established safety profiles. This ensures a higher level of confidence in their translational potential, maximizing the feasibility of applying this strategy in clinical settings.

To further improve the efficacy of selected drugs, encapsulating them into nanoparticles present a promising strategy. The advent of nanomedicine has introduced new possibilities for enhancing drug delivery and therapeutic efficacy. Nanomedicines utilize the EPR effect to achieve passive targeting of chemotherapy drugs to tumors, thereby reducing systemic toxicity and increasing drug accumulation at the tumor site. The continuous development of various nanomaterials has contributed to the evolution of nanomedicine (23). Encapsulating drugs in nanoparticles can help address several limitations associated with free drugs (24), thereby improving their therapeutic performance. Polymeric vesicles, a type of nanocarriers, have gained attention in cancer therapy due to their versatile properties, such as customizable composition, size, surface characteristics, and membrane properties (25). These vesicles can be designed to optimize interactions with therapeutic agents based on their physicochemical properties (26). A key benefit of polymeric vesicles is their ability to leverage the redox differences between the tumor cell’s intracellular and extracellular environments Tumor cells typically have much higher levels of reduced glutathione (GSH) inside the cell-ranging from 10 mM- compared to the extracellular space, where GSH concentrations are much lower (10–100 nM). This gradient arises due to enzymes like NADPH and GSH reductase, which reduce oxidized glutathione (GS-SG) to its active, reduced form GSH within the cytoplasm and various organelles, including mitochondria and the nucleus. Furthermore, many tumor tissues exhibit elevated GSH levels, often four times higher than those in normal cells (27). This redox disparity can be utilized to develop drug-delivery vesicles that are sensitive to the high GSH concentration in tumor. By incorporating disulfide bonds into the structure of these vesicles, drug payloads can be specifically released in response to the reductive conditions found in the tumor microenvironment (28–30).

**Figure 1:**
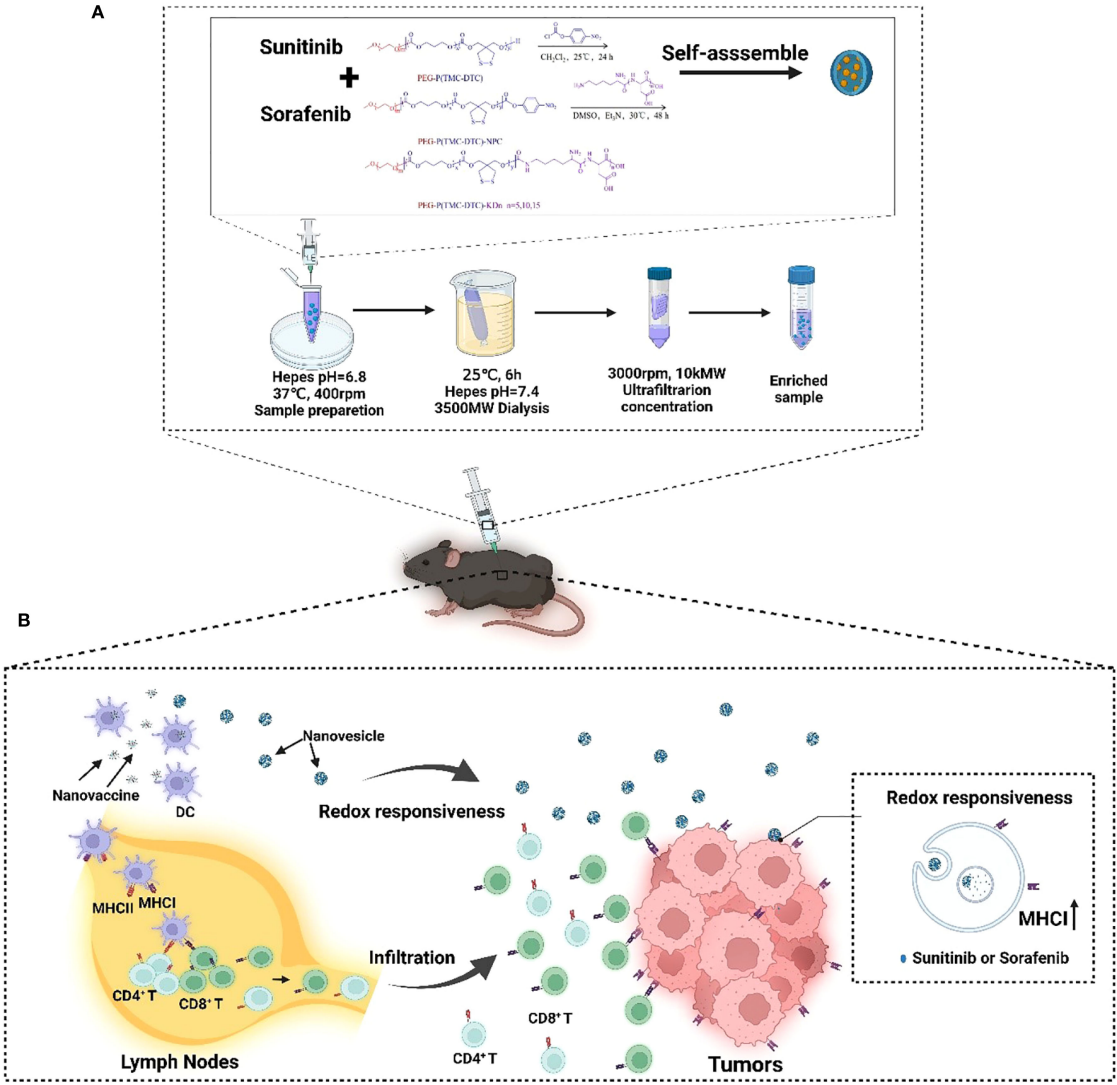
A diagrammatic explanation of how the integration of nanovaccines and nanovesicles can effectively target and destroy cancer cells through a synergistic mechanism. Once injected subcutaneously, nanovaccines are captured by APCs in the draining lymph nodes. These APCs trigger the activation of T cells, which are primed to recognize and attack tumor cells. As these T cells travel to the tumor site, they initiate the destruction of cancer cells. In parallel, the nanovesicles deliver KTIs to the tumor cells, boosting the expression of MHC I on their surfaces. This enhanced MHC I expression leads to more effective recognition of tumor cells by tumor-specific T cells, which in turn elevates the effectiveness of anti-tumor immunotherapy. **(A)** The process of preparing nanovesicles loading TKIs. **(B)** The mechanism of improving the efficacy of cancer nanovaccines by elevating the expressions of MHC I.

Although significant progress has been made in monotherapies and conventional cancer treatments, improving therapeutic efficacy remains a major challenge. To address these challenges, combination therapies have emerged as a promising strategy. Once such approach involves cancer vaccines based on whole tumor antigens, which have shown considerable potential in preclinical models (31). We have previously developed a universal method for creating whole-tumor antigen-based nanovaccines, demonstrating their effectiveness in melanoma, lung, and breast cancer models (32). Herein, in this study, we introduce a novel strategy combining reduction-responsive nanovesicles loaded with Sunitinib or Sorafenib with whole-tumor antigen-loaded nanovaccines (**Figure 1**). This combination approach aims to overcome the constraints of monotherapy by enhancing both drug delivery and immune activation. By integrating the biodegradable, redox-responsive nanovesicles (SUN-KD10 and SOR-KD10) with whole-tumor antigen nanovaccines, our goal is to improve therapeutic outcomes through more effective tumor targeting and activation of the immune system. This combined strategy offers a comprehensive solution to enhance the overall efficacy of cancer immunotherapy, providing a multifaceted approach to tumor treatment.

## Methods

### Reagents

The GSH used in this study, with a purity of 99%, was obtained from Shanghai Yuanye Co., Ltd. The Poly (lactic-co-glycolic acid) (PLGA)(MW24,000-38,000 Da, Cat. No. 719870; MW7,000 to 17,000 Da, Cat. No. 719897), and poly (vinyl alcohol) (PVA) (MW 9,000-10,000 Da, Cat. No. 360627), were purchased from Sigma Aldrich. The Poly (I: C) (vac-pic) material came from InvivoGen. In addition, we obtained Coumarin and LysoTracker™ Red from Beijing Solable Technology Co., Ltd. The KD_10_ peptide (KDDDDDDDDDD, >99% purity) was obtained from ChinaPeptides Co., Ltd. (PEG-P(TMC-DTC)-KD10 conjugate was prepared by the research group led by Professor Meng Fenghua at the Laboratory of Materials and Chemical Engineering. Shanghai Bio-Tech Biotechnology Co., Ltd. supplied us with Hoechst 33342, and we acquired the CpG oligonucleotides from Shanghai Sangon Biotech Co. Ltd. The drugs Sorafenib, Sunitinib, Lenvatinib, Ibrutinib, Azacytidine (5-Aza), Decitabine (DAC), Chloroquine (CQ), Doxorubicin (Dox), and Losartan were acquired from Shanghai Aladdin Co. Ltd.

### Ethics of animal studies

All animal procedures were conducted in accordance with the ethical guidelines approved by the Animal Care and Use Committee at Soochow University. All mice were kept in a specific pathogen-free (SPF) facility, conditions at 22 ± 1°C, 50 ± 10% humidity, with a 12-hour light/dark cycle and continuous ventilation.

### Cells

The melanoma cell line (B16F10) and the breast cancer cell line (4T1) were sourced from the Cell Bank of the Chinese Academy of Sciences in Shanghai, China.

B16F10 cell were maintained in Dulbecco’s Modified Eagle Medium with high glucose (DMEM hi) and 4T1 cells in RPMI 1640, both supplemented with 10% fetal bovine serum (FBS) and 1% penicillin-streptomycin (P/S).

### Screening of MHC I up-regulation drugs

B16F10 and 4T1 cell lines serve as valuable models for screening compounds that upregulate MHC I expression. Cells in the logarithmic growth phase were seeded at1.0×10^5^ cells per well in 24-well plates and incubated at 37°C until reaching approximately 60% confluence. The cells were then treated with varying concentrations (0, 1.25 M, 2.5, 5.0, and 10 µM) of Sorafenib, Sunitinib, Lenvatinib, Ibrutinib, 5-Aza, DAC, CQ, or Dox for 24 hours. Following treatment, cells were harvested, stained with a live/dead viability kit, and blocked with Fc receptor blocker to minimize non-specific binding. Subsequently, the cells were labelled using PE-conjugated anti-mouse H-2Kb antibody (MHC I, Clone AF6-88.5, Biolegend). MHC I expression was assessed using FACSAria™ III flow cytometer, followed by analysis of the results using FlowJo 10 software.

### Synthesis of PEG-P(TMC-DTC)-KD_10_

The synthesis of PEG-P(TMC-DTC)-KD_10_ followed the protocol outlined in our earlier work (33). Firstly, the terminal hydroxyl group of PEG-P(TMC-DTC) (molecular weight range 5.0-(14.9-2.1) kg/mol and a polydispersity index (PDI) Mw/Mn = 1.1) was functionalized by activation with p-nitrophenyl chloroformate (p-NPC). Following the activation, the modified polymer was then reacted with the KD_10_ peptide.

In the initial stage, 1.0 g (equivalent to 45.5 μmol) of PEG-P(TMC-DTC) was dissolved in 10 mL of dichloromethane (DCM) under a nitrogen atmosphere. Pyridine (18.0 mg, 227.8 μmol) was added, and the mixture was cooled to 0 °C. A solution of p-NPC (48.4 mg, 240.3 μmol) in 1.0 mL of DCM was gradually added dropwise over 30 minutes. The reaction proceeded at r.t. for 24 hours. The resulting PEG-P(TMC-DTC)-NPC intermediate was obtained in 90% yield via filtration, precipitation in cold diethyl ether, and vacuum drying.

Next, 916.6 mg (41.7 μmol) of the resultant PEG-P(TMC-DTC)-NPC was dissolved in 9 mL of anhydrous dimethyl sulfoxide (DMSO) under nitrogen. This solution was then gradually added over 30 minutes to a stirred mixture of KD_10_ peptide (60.0 mg, 83.3 μmol) and triethylamine (4.2 mg, 41.7 µmol) in 4 mL of DMSO. The reaction was maintained at 30 °C for 48 hours. The product was purified by dialysis (MWCO 3500 Da) against DMSO for 36 hours, then against DCM for 6 hours. The resulting solution was concentrated to ~100 mg/mL by rotary evaporation, followed by precipitation with cold diethyl ether, filtration, and vacuum drying to yield PEG-P(TMC-DTC)-KD_10_ in 91% yield.

The conjugation of the KD_10_ peptide was verified by HPLC, using a mobile phase composed of 150 mM phosphate buffer (PB) (pH 7.0) mixed with acetonitrile at a ratio of 90:10, UV detection at 214 nm, and a column temperature of 30 °C.

### Preparation of self-crosslinking drug-loaded nanovesicles

Self-crosslinking drug-loaded nanovesicles were successfully fabricated via the solvent exchange procedure. Specifically, Sunitinib or Sorafenib(10–20 wt.%, ~10 mg/mL), and KD_10_(5–20 wt.%, ~40 mg/mL), both dissolved in DMSO, were mixed (total volume:100 μL) and added dropwise to 0.9 mL of HEPES buffer (pH of 6.8) under continuous stirring at 400 rpm. After the addition was complete, mixture was allowed to rest for 3 minutes and then incubated overnight at 37°C.

The following day, the resulting nanovesicles were placed into a dialysis bag (MWCO 35 kDa) and dialyzed against in HEPES buffer (pH 7.4) for 6 hours, with hourly buffer changes to eliminate unencapsulated substances. Final purification was performed via ultrafiltration using a 10 kDa MWCO centrifugal filter, yielding the purified drug-loaded nanovesicles.

### Collection of tumor tissue lysates

Tumor tissues from B16F10 (C57BL/6 mice) or 4T1 (BALB/c mice) models were first minced into small fragments. These were then incubated in ultrapure water, following our previously published protocols (31, 32, 34, 35). The mixture underwent several freeze-thaw cycles, followed by, sonication to ensure complete cell disruption. Lysates underwent centrifugation at 12,000 rpm for 5 minutes to separate soluble and insoluble fractions. The supernatant, containing water-soluble components, was carefully collected for further use. In contrast, the pellet, comprising the water-insoluble components, was re-suspended in 8 M urea to solubilize the proteins.

### Preparation of nanovaccine A encapsulating water-soluble components

Nanovaccine A was prepared using a double-emulsion method, as detailed in our previous research (31, 32, 34–37). Initially, an aqueous solution containing water-soluble components was prepared in endotoxin-free water at a total concentration of 80 mg/mL, including 10 mg/mL poly (I: C) and 1mg/mL CpG1018. A 200 μL aliquot of this solution was added to 1 mL of PLGA dissolved in dichloromethane (100 mg/mL) and sonicated for 1 minute to form the primary emulsion. The primary emulsion was then transferred into 2.5 mL of PVA solution (20 mg/mL) and sonicated for 45 seconds to generate the double emulsion. This was gradually added to 50 mL of PVA solution (5 mg/mL) and stirred at r.t. for 4 hours to facilitate the solidification of nanoparticles. Nanoparticles were collected by centrifugation at 12,000 rpm for 25 minutes, re-dispersed in 10 mL of 4% trehalose solution, and lyophilized for 48 hours to obtain the final dried product.

### Preparation of nanovaccine B encapsulating water-insoluble components

The preparation of nanovaccine B followed the same procedure as nanovaccine A, with the only difference being the replacement of the water-soluble component with a water-insoluble component in 8 M urea (80 mg/mL), containing 10 mg/mL poly (I: C) and 1mg/mL CpG1018.

### Characterization of the nanovesicles/nanovaccines

Nanovesicles and nanovaccines were extensively characterized based on multiple parameters, including size, zeta potential, morphology, and drug-loading efficiency. Particle size distribution and zeta potential were measured using dynamic light scattering (DLS) with a Zetasizer Nano-ZS (Malvern Instruments, Worcestershire, UK). Morphological characteristics were examined via transmission electron microscopy (TEM) using a Hitachi HT7700 instrument. Drug loading capacity (DLC) and drug loading efficiency (DLE) were quantified using UV spectrophotometry and calculated formulas follows:

DLC (wt.%) = (amount of drug/total amount of drug and polymer) × 100
DLE (%) = (measured drug dose/theoretical drug dose) ×100

The encapsulation efficiency (EE) of nanovaccines was determined using a Nanodrop spectrophotometer and calculated as:

EE (%) = (protein load/protein input) × 100%

### *In vitro* release of sunitinib and sorafenib

The reduction-responsive characteristics of the nanovesicles (Sun-KD10 and Sor-KD10) were assessed by exposure to GSH. Specifically, 1 mL of nanovesicle suspension (200 µg/mL) was mixed with 10 mM GSH under a nitrogen atmosphere. The mixture was incubated at 37°C in a shaker set to 200 rpm. Particle size and polydispersity index (PDI) were monitored over time using DLS.

To assess the *in vitro* release of Sunitinib and Sorafenib from the nanovesicles, 1 mL of Sun-KD10 and Sor-KD10 (200 µg/mL) was separately loaded into dialysis bags (MWCO 35 kDa). These bags were immersed in 25 mL of HEPES buffer (pH 7.4) containing 5 mM HEPES and 0.2% Tween 80, with or without the addition of 10 mM GSH. The system was maintained at 37°C and agitated at 200 rpm. At predetermined time points, 5 mL of the release medium was withdrawn and replaced with an equal volume of fresh buffer to maintain constant volume. The collected samples were lyophilized and redissolved in DMSO, and the concentrations of Sunitinib and Sorafenib were determined using UV spectrophotometry.

### Cytotoxicity assay (MTT assay)

B16F10 or 4T1 cells were retrieved and seeded (at a density of 1.5 × 10³ cells/well) in low-adhesion 96-well plates. After 4 hours treatment with PEG-P(TMC-DTC)-KD10 (0.1 to 1.0 mg/mL), cells were incubated for 44 hours before MTT assay. Absorbance at 570 nm was measured to determine viability.

### *In vitro* cellular uptake of nanovesicle in B16F10/4T1 tumor cells

Both B16F10 and 4T1 cells were plated in low-adhesion 96-well plates at a density of 5.0 × 10^5^ cells per well and incubated overnight to allow adhesion and expansion. The following day, Co6-KD10 nanovesicles were added and incubated for 4 hours. Cellular uptake was then assessed by flow cytometry using FACSAriaTM III. Data were analyzed using FlowJo 10 software.

### Payload release and endosomal release mechanisms

To investigate the release of contents from nanovesicles and nanoparticles and their capacity to escape endosomes, experiments are conducted using B16F10, 4T1, and DC2.4 cell lines. Cells were seeded at a density of 5.0 × 10^4^ cells per well in glass-bottom dishes and incubated overnight to allow adhesion and expansion. To ensure proper attachment, the cells were left to adhere overnight at 37°C with 5% C_2_. The following day, nanovesicles and nanoparticles loaded with Coumarin 6 were added to cells and DC2.4, respectively, at appropriate concentrations and incubated for 6 hours to facilitate internalization. To visualize endosomes, LysoTracker™ Red was added 0.5–2 hours before fixation. Cells were then fixed with 4% paraformaldehyde and stained with Hoechst for 15 minutes to label nuclei. After repeated washing to remove unbound materials and excess dye, cells were mounted using VECTASHIELD anti-fade medium containing DAPI. Cargo release and endosomal escape were examined using confocal microscopy.

### Assessment of MHC I expression on the surface of mouse tumor cells following co-incubation

B16F10 or 4T1 cells (1.0 × 10^5^ cells per well) were seeded in low-adhesion 12-well plates and incubated at 37°C for 20 hours until reaching approximately 60% confluence. The cells were then treated with Sunitinib, Sorafenib, SUN-KD10, or SOR-KD10, and co-cultured for an additional 24 hours. After incubation, cells were collected, stained with a live/dead viability dye, and treated with an Fc receptor blocker to minimize non-specific binding. Surface MHC I expression was assessed by staining with PE-anti-mouse H-2Kb (MHC I, Clone AF6-88.5, Biolegend) followed by flow cytometry using FACSAriaTM III. Data were analyzed using FlowJo 10 software.

### Therapeutic efficacy of nanovesicles or/and nanovaccines

The experimental procedures for establishing cancer models and treatment schedules were standardized across all studies. In the TNBC mouse model, 4.0×10^5^ 4T1 cells were injected subcutaneously (s.c.) into the right flank of BALB/c mice on day 0. For the melanoma mouse model, C57BL/6 mice were injected with 1.5 × 10^5^ B16-F10 cells.

Mice in the nanovaccine-only groups received 2 mg doses of nanovaccine A and nanovaccine B (ratio 1:1) on days 4, 7, 10, 15, and 20. In groups receiving both nanovaccine and free drugs, the free drugs were administered daily, starting on day 6. For groups treated with nanovaccines combined with nanovesicles, SUN-KD10 or SOR-KD10 was administered every other day, beginning on day 6. Control mice received an equivalent volume of sterile PBS in place of the treatments.

Starting on day 0, the tumor volume measurements were taken every 3 days. Detailed dosing schedules and concentration information for combination treatments are provided in **Supplementary Table S3**.

### Tumor volume measurements

Tumor size was assessed using calipers, by measuring two perpendicular diameters. Tumor volumes (V) were calculated using the formula V = 0.52 × a × b^2^, where ‘a’ is the larger diameter and ‘b’ is the smaller diameter. Mice were humanely euthanized once tumor volume reached 2000 mm^3^.

### Histopathology examination

Following humane euthanasia, major organs were harvested and fixed in in formalin at room temperature (r.t) for 48-hours. After fixation, tissues were processed for paraffin embedding. The paraffin-embedding samples were sectioned into 3 μm slices, mounted on glass slides, and air-dried. H&E staining was then performed, and the slides were examined under a microscope.

### Analysis of peripheral tumor-specific T cells

Splenocytes were harvested from each group 18 to 22 days after tumor cell inoculation and stimulated with nanovaccines for 48 hours. After stimulation, cells were incubated with live/dead viability dye for 30 minutes, followed by a 5-minute treatment with Fc receptor blocker. To identify T cell subsets, surface staining for CD3, CD8, and CD4 was performed, followed by intracellular staining for IFN-γ. Derails of all antibodies used for flow cytometry are provided below in **Table 1**:

**Table 1:** Antibodies used for low cytometry and their specifications.

Antibody	Clone	Fluorophore	Vendor	Volume
ZombieViolet™FixableViabilityKit		BV421	Biolegend	1.0uL
FcRBlock(CD16/32)	93		Biolegend	2.0uL
CD3	17A3	APC/cy7	Biolegend	1.5uL
CD4	GK1.5	PE/cy7	Biolegend	1.5uL
CD8a	53-6.7	PE	Biolegend	1.5uL
IFN-gamma	XMG1.2	APC	Biolegend	2uL

### Statistical analysis

All statistical analyses were performed using GraphPad Prism, version 8.3.0. Data are presented as mean ± standard deviation (SD) or standard error of the mean (SEM). Statistically significant was defined as *p < 0.05; **p < 0.01; ***p < 0.001. Asterisks indicate comparisons with the control group unless otherwise specified.

### Availability of data and materials

The materials that substantiate the claims made in this investigation are available in the principal article and its annexed files. Should you need more associated data, please contact the corresponding author for reasonable access.

## Results

### Identification of drugs that enhance MHC I expression on tumor cells

The process of MHC I antigen presentation is intricate and controlled by various signaling pathways, offering opportunities to pharmacologically target these pathways to restore MHC I expression on tumor cells. This strategy focuses on boosting the activation of these pathways to induce MHC I expression in cancer cells. In addition to the IFN-γ/STAT1 signaling pathway, which plays a key role in regulating MHC I expression, the hypermethylation of MHC I and related antigen-presenting genes reduces antigen presentation by inhibiting transcription. As a result, using DNA methyltransferase inhibitors can enhance the expression of tumor-associated antigens, thereby improving the efficacy of immunotherapy (38).

This study investigates the impact of several clinically used drugs on the expressions of MHC I molecules, including tyrosine kinase inhibitors (TKIs), which enhance MHC I expression through the activation of IFN-γ/STAT1 signaling pathway in cancer cells. We also examined the effects of epigenetic regulators, such as 5-Azacytidine (5-Aza) and decitabine (DAC), which promote MHC I expression by inhibiting DNA methylase. In addition, we assessed chloroquine (CQ) and doxorubicin (Dox) for their potential to increase MHC I levels on the surface of cancer cells.

**Figure 2:**
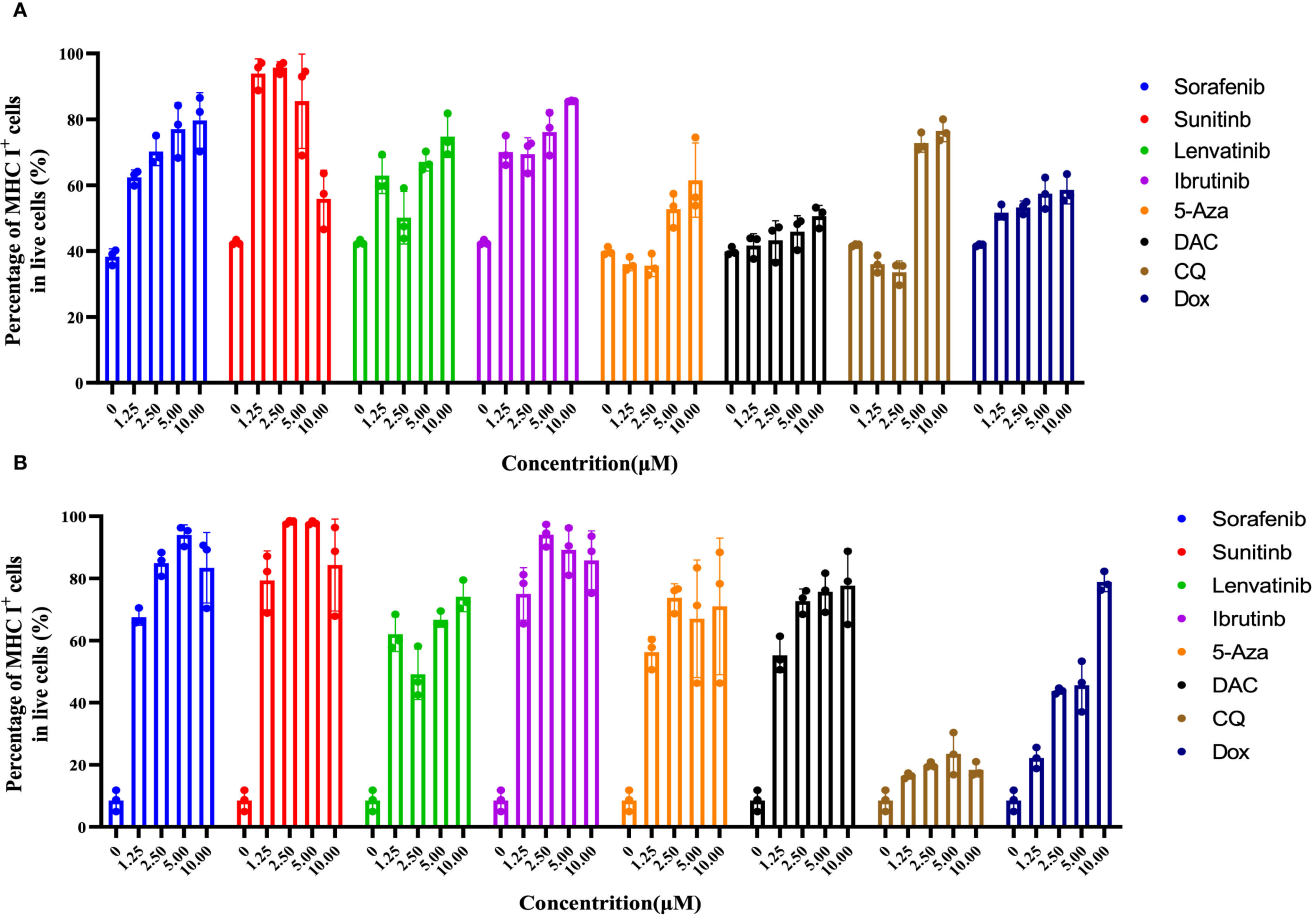
The combination of Sorafenib, Sunitinib, Lenvatinib, Ibrutinib, 5-Aza, DAC, CQ, and Dox promotes upregulation of MHC I expression on the membrane of B16F10/4T1 cancer cells during co-incubation. **(A)**, The effect of different drug concentrations (0 to 10 μM) on MHC I expression in B16F10 cells was examined by performing flow cytometric analysis after a 24-hour co-incubation period. **(B)**, Evaluation of MHC I expression on 4T1 cells using flow cytometry techniques post 24-hour exposure to different drug concentrations. The data are presented as the mean ± S.D. (n = 3).

To evaluate the impact of various drugs on MHC I expression, B16F10 cells were treated with drugs (ranging from 0 to10 μM) for 24 hours, and MHC I expression on the cell surface was analyzed (**Figures 2A, B**, **Supplementary Figure S1**). Approximately 40% of B16F10 cells expressed MHC I, which aligns with previous studies (39). Co-incubation with Sorafenib and Ibrutinib led to a dose-dependent increase in MHC I expression, reaching up to 90%. Sunitinib also promoted MHC I expression at lower concentrations though at 5 μM, a slight decrease in MHC I levels was observed, likely due to cytotoxicity. Higher concentration of 5-Aza, Lenvatinib, and CQ proved more effective in boosting MHC I expression on B16F10 cell. In contrast, Dox and DAC induced MHC I up-regulation at higher concentrations, but their effects were less pronounced compared to other drugs at similar doses (**Figure 2A**).

In contrast to melanoma cells, 4T1 breast cancer cells exhibited much lower MHC I expression (~15%) on their surface, likely due to the immune evasion properties of this ‘col’ tumor type. To evaluate the efficacy of the drugs in this context, 4T1 cells were co-cultured with drugs ranging from 0 to 10 μM for 24 hours and MHC I expression was assessed. The results mirrored those observed in B16F10 melanoma cells. Sorafenib, Sunitinib, and Ibrutinib effectively up-regulated MHC I expression at lower concentrations, while 5-Aza and Lenvatinib showed greater efficacy at higher doses. Dox and DAC also enhanced MHC I levels with increasing concentrations, though their effectiveness was lower than that of other drugs at similar doses. Interestingly, CQ did not significantly increase MHC I expression on 4T1 cells surface (**Figure 2B**). These findings suggest that TKIs, particularly Sorafenib, Sunitinib, and Lenvatinib, are more potent in boosting MHC I expression compared to other tested drugs. Based on these promising results, Sorafenib and Sunitinib were selected for combination therapy with cancer vaccines.

### Development of redox-sensitive nanovesicles encapsulating sunitinib and sorafenib

**Figure 3:**
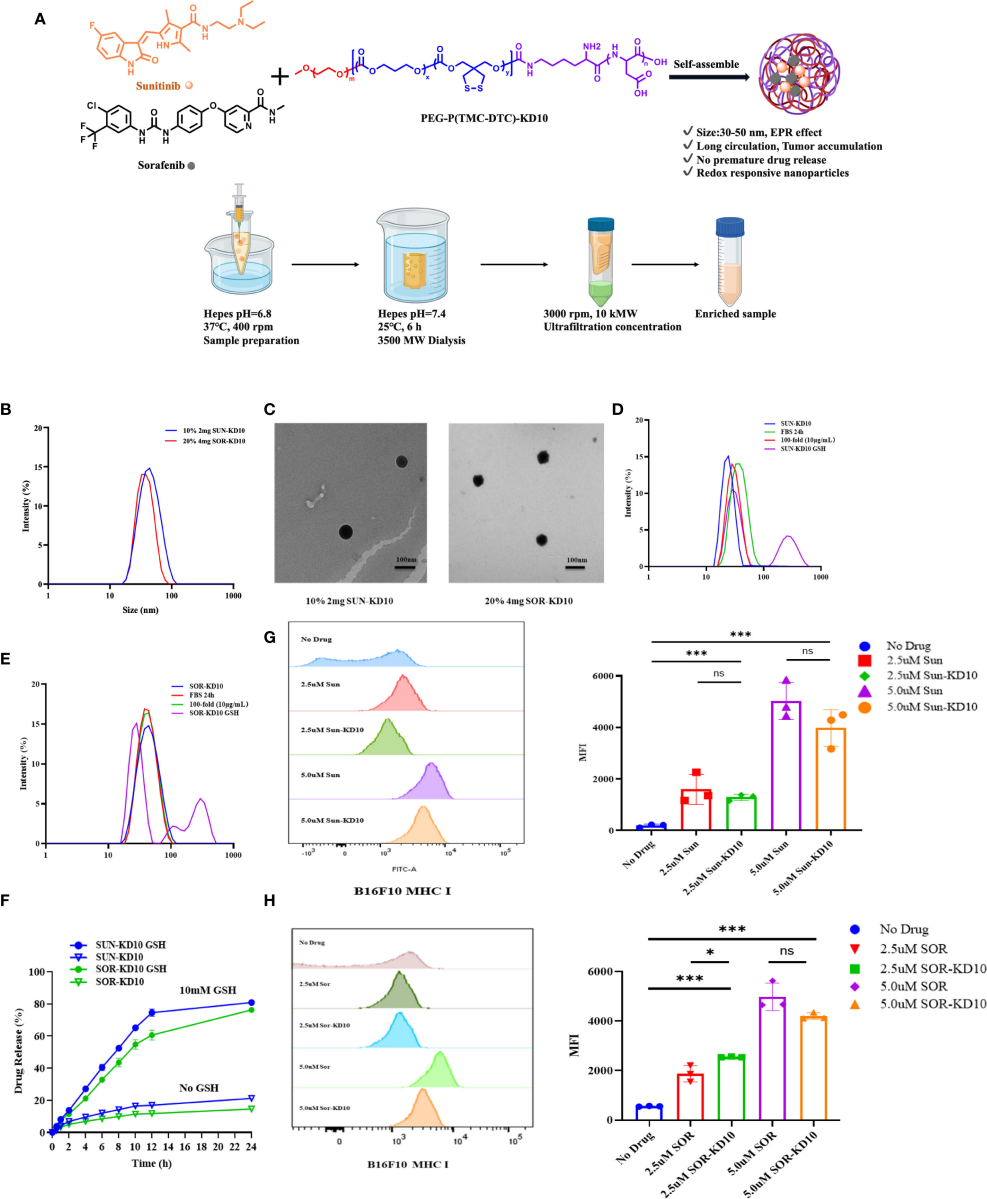
Fabrication and characterization of nanovesicles encapsulating sunitinib or sorafenib. **(A)** Preparation process of nanovesicles. **(B)** Particle size distribution of Sun-KD10 and Sor-KD10. **(C)** TEM images of Sun-KD10 and Sor-KD10 nanovesicles. The particle size distribution of Sun-KD10 **(D)** or Sor-KD10 **(E)** was analyzed following dilution (final concentration: 10 mg/L) in HEPES buffer (pH 7.4, 5 mM) containing10% FBS and GSH to mimic the reducing conditions of the tumor microenvironment. **(F)** Drug release profiles of Sunitinib and Sorafenib from Sun-KD10 and Sor-KD10 were evaluated in simulated intracellular GSH-rich reducing environments (HEPES, pH 7.4, 5 mM) at 37°C (n=6). **(G, H)** The impact of SUN-KD10 and SOR-KD10 on MHC I expression on B16F10 cells after 24 hours of co-incubation was assessed. Flow cytometry histograms displayed the peak and mean fluorescence intensity (MFI) of MHC I expression in B16F10 cells after 24 hours of co-incubation with SUN-KD10 and SOR-KD10. * indicates p<0.05, *** indicates p<0.005, ns means no significant.

To enable efficient and targeted delivery of TKIs to tumor cells, we developed two types of biodegradable nanovesicles, Sun-KD10 and Sor-KD10, using reversible disulfide cross-linking. These nanovesicles were prepared via the solvent exchange method (33, 40, 41) (**Figure 3A**, **Supplementary Figures S2**, **S3**). Previous studies (33, 41) have shown that disulfide bonds significantly improve the stability of the nanovesicles in physiological conditions. These bonds are responsive to the reduced environment found at the tumor site, facilitating the controlled release of the encapsulated drugs. The PEGylated surface of the nanovesicles enhances their circulation time and reduces off-target drug accumulation. Furthermore, the negatively charged KD10 inside nanovesicles interacts electrostatically with the amino groups of sunitinib or sorafenib in an acidic environment. This design ensures that drug release is precisely targeted at the tumor site, optimizing therapeutic efficacy while minimizing systemic toxicity.

### Characterization of nanovesicles encapsulating sunitinib or sorafenib

To explore the encapsulation efficiency and drug loading capacity of nanovesicles, Sun-KD10 or Sor-KD10 were prepared using different concentrations of PEG-P(TMC-DTC)-KD10 (2 mg/mL or 4 mg/mL) and varying theoretical drug loadings (10%, 20%). The results, shown in **Supplementary Table S1**; **Figure 3** demonstrate that Sun-KD10 prepared with 2 mg/mL PEG-P(TMC-DTC)-KD10 and a 10% theoretical drug load achieved a loading capacity of 6.57% and an encapsulation efficiency of 63.25%. Similarly, Sor-KD10, prepared with 4 mg/mL PEG-P(TMC-DTC)-KD10 and a 20% theoretical drug load, exhibited a drug loading capacity of 4.73% and an encapsulation efficiency of 40%. The particle sizes of Sun-KD10 and Sor-KD10 were measured at 45.17 ± 0.86 nm and 40.11 ± 0.16 nm, respectively (**Figure 3B**). TEM analysis demonstrated that both nanovesicles possess spherical morphologies (**Figure 3C**). These findings suggest that Sun-KD10 and Sor-KD10 have high drug loading capacities, efficient encapsulation, small particle sizes, and excellent dispersion, which enhance their accumulation at tumor sites through the enhanced EPR effect.

To assess the stability and redox-responsiveness of Sun-KD10 and Sor-KD10, their particle sizes were measured after 100-fold dilution in HEPES buffer (pH 7.4, 5 mM) or incubation in FBS for 24 hours. The results revealed no significant changes in particle size or PDI under these conditions, indicating that both nanovesicles exhibit good stability in physiological conditions (**Figures 3D, E**). To simulate the tumor’s reducing environment, Sun-KD10 and Sor-KD10 were exposed to GSH. GSH disrupts the disulfide bonds, converting them into sulfhydryl groups and increasing the hydrophilicity of the nanovesicles. This change caused the nanovesicles to swell rapidly within 2 hours, resulting in increased particle sizes and broader particle size distributions, reflecting their redox-responsiveness (**Figures 3D, E**). Additionally, the drug release profiles of Sun-KD10 and Sor-KD10 were evaluated in various media at different time points. As shown in **Figure 3F**, in HEPES buffer (pH 7.4, 5 mM) without GSH, the compact structure of the cross-linked nanovesicles and the strong π-π interactions between the drug and tyrosine benzene rings resulted in slow drug release, with less than 20% cumulative release over 24 hours. However, in the presence of 10 mM GSH, the nanovesicles responded quickly, achieving 50% cumulative release within 8 hours and 80% within 24 hours.

Additionally, the cellular uptake of nanovesicles in tumor cells was investigated using flow cytometry. First, the cytotoxicity of the nanovesicles was assessed via MTT assay after incubating them with B16F10 and 4T1 cells. The results showed that the nanovesicles were non-cytotoxic within 0.1-1.0 mg/mL (**Supplementary Figure S4**). The uptake of nanovesicles by both B16F10 and 4T1 cells exhibited a dose-dependent increase, reaching a maximum after 4 hours (**Supplementary Figure S4**). Confocal microscopy further confirmed that nanovesicles uptake by both cell types reached maximum levels at 4 hours (**Supplementary Figure S4**). These findings demonstrated that tumor cells effectively internalize the nanovesicles, which is essential for their therapeutic potential.

### Nanovesicles encapsulating sunitinib or sorafenib enhance MHC I expression on tumor cell surfaces

To assess the effect of nanovesicles on MHC I expression on tumor cell surfaces, B16F10 cells were treated with nanovesicles, and the MHC I levels were then assessed. As illustrated in **Figures 3G, H**, both SUN-KD10 and SOR-KD10 significantly increased MHC I expression on B16F10 cells compared to free Sunitinib and Sorafenib. The enhancement of MHC I expression was more pronounced with higher concentrations of the loaded drugs. Notably, the ability of SUN-KD10 and SOR-KD10 to upregulate MHC I expression was similar to that of the free drugs at equivalent concentrations, indicating that encapsulating Sunitinib and Sorafenib in the nanovesicles did not compromise their ability to modulate MHC I expression.

### Preparation and characterization of whole-tumor antigen nanovaccines

Effective cancer vaccine development requires approaches that either enhance the pool of tumor-specific T cells from the naïve repertoire or reinvigorate inactive or suppressed tumor-specific T cells. The effectiveness of therapeutic vaccines largely depends on the specific characteristics of the antigens utilized. Due to the limited repertoire of well-characterized common cancer antigens and the inherent complexity and variability of cancer antigen profiles, a universal vaccine is not capable of meeting the diverse needs of all patients. Consequently, tumor tissues or cells are considered the most promising source of antigens for developing cancer vaccines (42). While water-soluble tumor antigens are frequently employed in vaccine development, the inclusion of water-insoluble antigens presents substantial challenges. Direct injection of tumor tissue or cell lysates has shown only modest immune responses and has not demonstrated significant therapeutic benefit (42).

To overcome these challenges, we developed whole-tumor antigen nanovaccines (VAC) (32). By using 8M urea, we solubilized the water-insoluble components of tumor tissues or cells, enabling the VAC to incorporate both water-soluble and water-insoluble antigens. To improve uptake by APCs, the particle size was carefully optimized (**Supplementary Figure S5**, **Supplementary Table S2**). Furthermore, co-loading of Poly (I: C) and CpG1018 enhanced the VAC capacity to activate tumor antigen-specific T cells following taken up by APCs.

Confocal microscopy analysis demonstrated that NPs/MPs payloads were presented in a cross-presentation way via both the MHC I and MHC II pathways. (**Supplementary Figure S7c**).

### SUN-KD10/SOR-KD10 enhances the therapeutic effectiveness of nanovaccines in melanoma treatment

**Figure 4:**
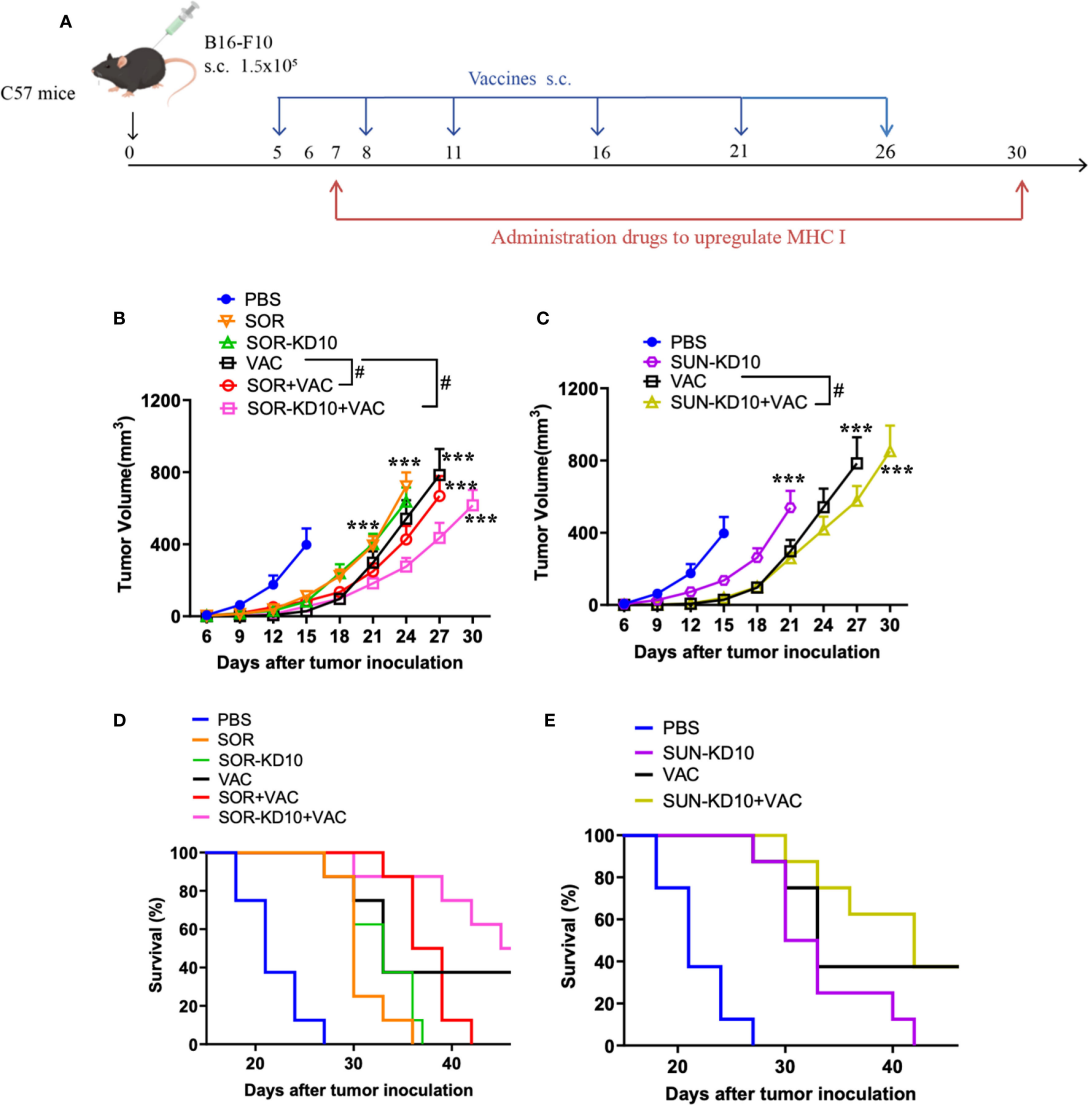
Study on the therapeutic impact of nanovaccines and nanovesicles on B16F10 melanoma using a mouse model (n≥7). **(A)** Schematic depicting the timing of tumor introduction and subsequent treatment phases. **(B, C)**, Inhibition of tumor development in melanoma-bearing mice was observed when treated with nanovaccines and nanovesicles. **(D, E)** Survival rates of melanoma-bearing mice treated with nanovaccines and nanovesicles. Data are presented as mean ± S.E.M., with statistical significance determined by using an unpaired t-test. *** indicates p<0.005 and # indicates p<0.05.

The preliminary evaluation of the therapeutic effects from the combination of nanovesicles and VACs was performed in a B16F10 melanoma mouse model. Following the inoculation of tumors, VACs were given five times, while drug-loaded nanovesicles or free drugs were introduced every other day starting from day 6 (**Figure 4**, **Supplementary Table S3**). Treatment with VAC, SUN-KD10, or SOR-KD10 led to a notable decrease in tumor size and an increase in lifespan when compared to the PBS control group. In addition, the group treated with VAC plus SOR-KD10 demonstrated enhanced therapeutic outcomes when compared to the VAC and SOR group alone, indicating the critical importance of nanovesicle encapsulation for chemotherapeutic agents. When VAC was given together with SUN-KD10 or SOR-KD10, it resulted in a notably better therapeutic effect compared to using VAC alone or each drug separately. These observations point to the potential of TKIs, such as Sunitinib and Sorafenib, when packaged in nanovesicles, to augment the efficacy of VACs and achieve a synergistic therapeutic response.

### SUN-KD10/SOR-KD10 enhances the therapeutic efficacy of nanovaccines in breast cancer treatment

**Figure 5:**
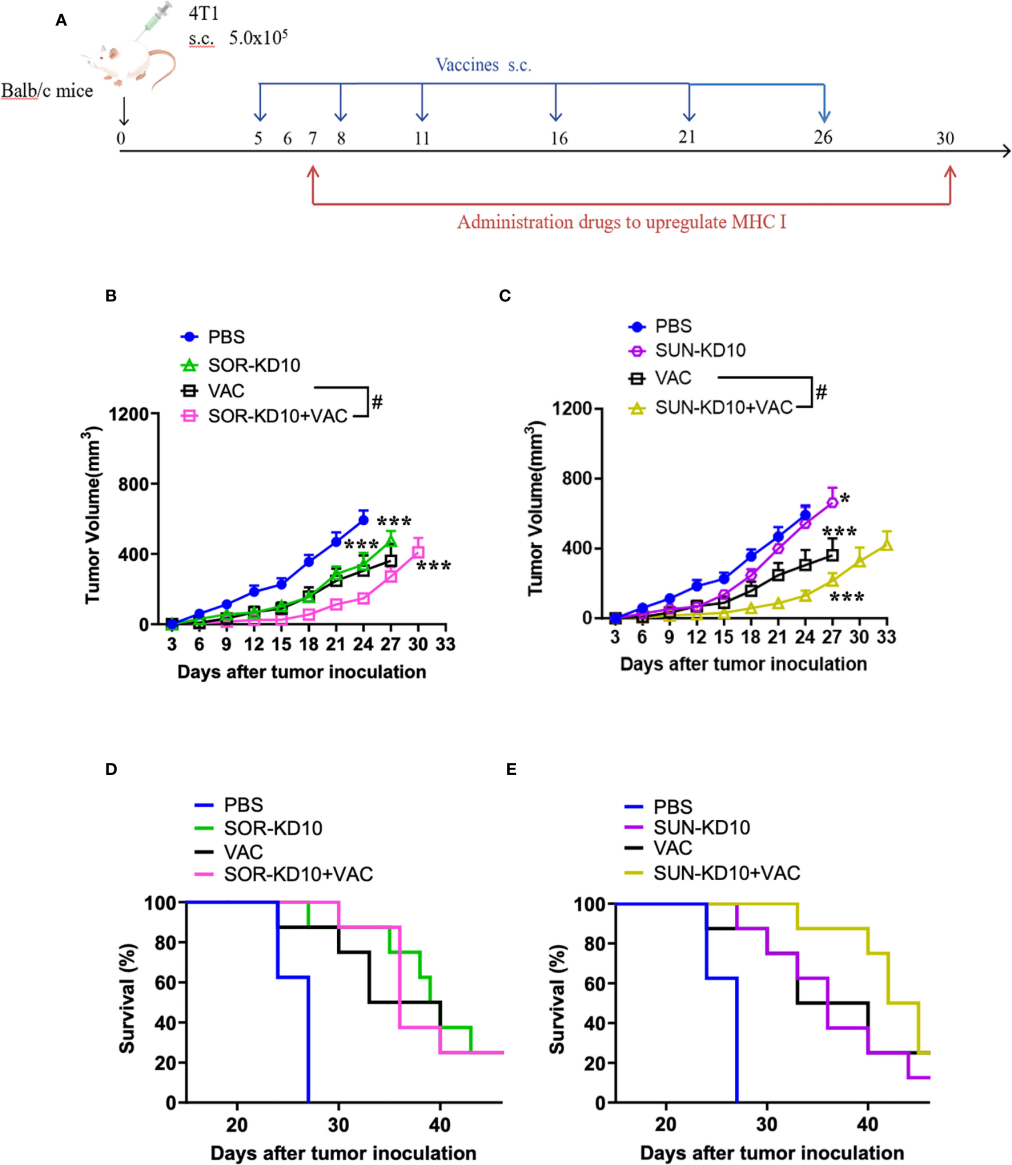
Assessment of therapeutic efficacy of nanovaccines and nanovesicles in TNBC 4T1 mouse model (n=8). **(A)** Diagram illustrating the timing and process of tumor engraftment alongside the treatment regimen. **(B, C)** Nanovaccines and nanovesicles demonstrated tumor growth suppression in mice with 4T1 tumors. **(D, E)** The survival benefits experienced by 4T1 tumor-bearing mice that received treatments involving nanovaccines and nanovesicles. Data are presented as mean ± S.E.M. and statistical significance was determined using an unpaired t-test. Asterisks denote significance levels: * indicates p<0.05, *** indicates p<0.005 and # indicates p<0.05.

To further verify the therapeutic efficacy in a cold-tumor model, the therapeutic efficacy of combining nanovesicles + VACs was evaluated in a **t**riple-negative breast cancer (TNBC) mouse model (**Figure 5**). Similar to the results observed in the melanoma model, treatment with VACs, SUN-KD10 or SOR-KD10 markedly delayed tumor progression compared to untreated group. Moreover, combining VAC with SUN-KD10 or SOR-KD10 led to a significant improvement in therapeutic efficacy. This combination achieved a synergistic effect, demonstrating the potential of merging cancer vaccines with TKIs. Findings from this cold tumor model further support the promising therapeutic potential of combining TKIs with VAC as a novel strategy.

### No adverse effects were detected throughout the treatment

Body weight measurements of the mice were recorded throughout the entire treatment duration, showing no significant alterations in the treatment groups, which suggests that there were no observable toxic effects (**Supplementary Figure S6**). In addition, the principal organs of the treated mice were obtained and examined. All examined organs from mice receiving diverse treatment approaches appeared normal (**Supplementary Figure S7**), implies the safety of the treatment as it shows no toxicity in the tested animals.

### An increase in tumor-specific T cells was observed in peripheral tissues after treatment

**Figure 6:**
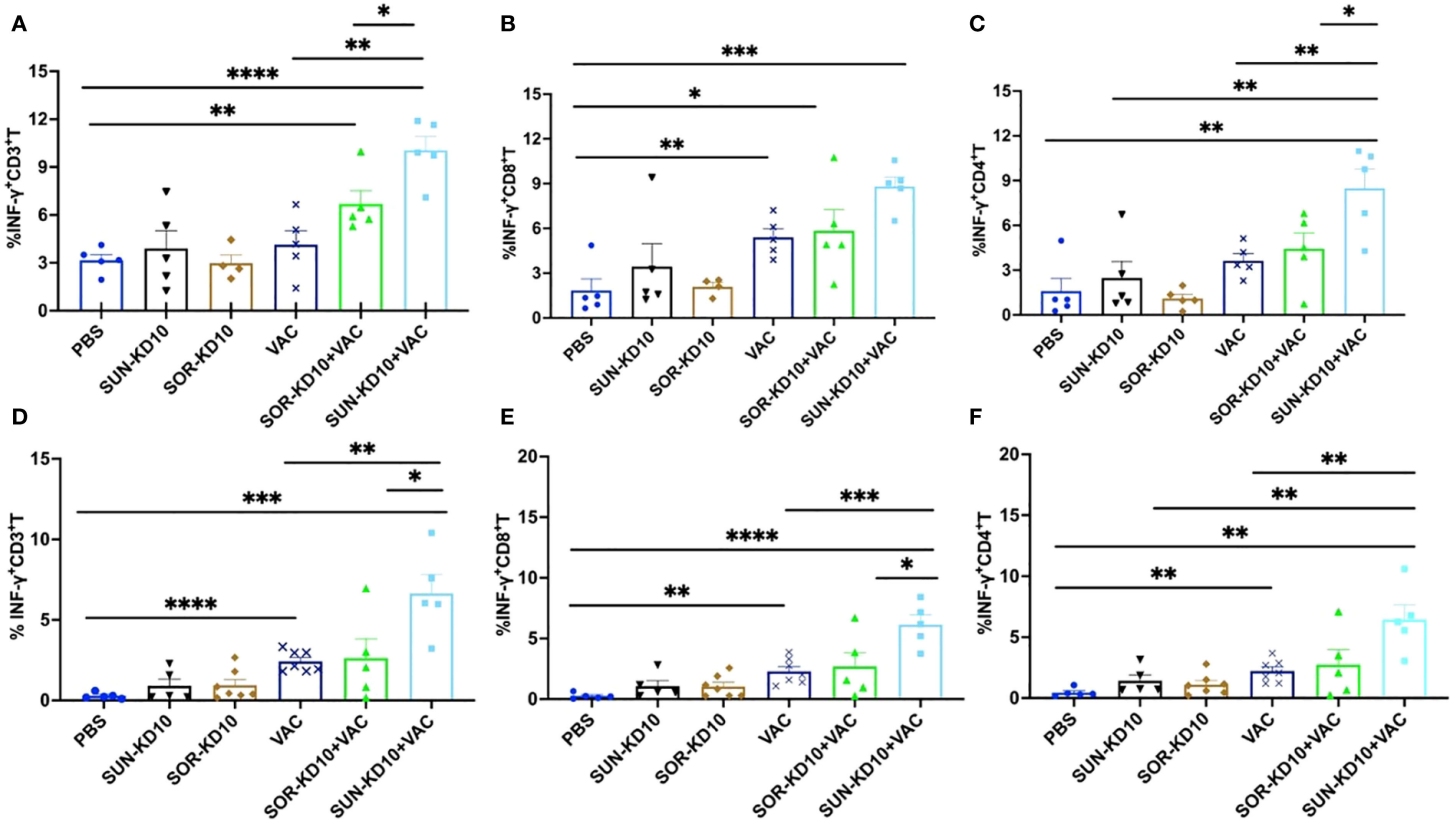
Examination of tumor-specific T cell populations in the spleenocytes and draining lymph nodes of breast cancer-bearing mice with tumors post-treatment. **(A–C)** Evaluation of tumor-specific T cells within the splenocytes (n=5). **(D–F)** Examination of tumor-specific T cells within the lymph nodes (n=5). Data are represented as mean ± S.D., with statistical significance determined using an unpaired t-test. * means p<0.05, ** means p<0.01, *** means p<0.001.

For assessing the development of tumor-specific T cells upon treatment, we assessed their abundance in the spleen and lymph nodes. To study the activation of tumor-specific T cells, nanoparticles carrying whole-tumor antigens employed for stimulation, followed by an analysis using flow cytometry techniques. The levels of CD3^+^INFγ^+^, CD8^+^INFγ^+^, and CD4^+^INFγ^+^ T cells in peripheral tissues were quantified as tumor-specific T cells following co-incubation with the antigen-loaded nanoparticles (**Figure 6**; **Supplementary Figure S8**). Data from our study showed that treatment with SUN-KD10/SOR-KD10 and VACs enhanced the activation and identification of tumor-specific T cells in the spleen and lymph nodes when stimulated by tumor antigens (**Figure 6**). Results from this study demonstrate that the combined application of SUN-KD10/SOR-KD10 and VACs greatly amplifies the generation of tumor-specific T cells in peripheral tissues, enhancing their potency to detect and eradicate tumor cells.

## Discussions and conclusions

The goal of cancer treatment is to shift the disease from being fatal to a manageable, chronic disease. Achieving this requires boosting immune surveillance and eliciting robust tumor-specific immune responses. Many different cancer immunotherapy, such as cancer vaccines play a crucial role by activating tumor-specific T cells that recognize tumor antigens presented via MHC I molecules (7, 10, 16, 32). However, downregulation or loss of MHC I is a main mechanism of immune evasions in solid tumors and is associated with poor prognosis and resistance to immunotherapy. If MHC I expressions on tumor cells could be elevated, pre-existing or newly activated tumor-specific T cells, such as activated by cancer vaccines, would recognize and attack tumor cells more easily (31, 34). Therefore, exploring efficient methods to elevate the expression of MHC I on cancer cells can be applied to treat cancer alone or applied with other cancer immunotherapy strategies. Such combinational treatment potentially can improve the therapeutic efficacy of cancers in clinic.

We previously developed whole-tumor-antigen nanovaccines capable of inducing broad-spectrum tumor-specific T cell responses by efficiently delivering a complex repertoire of tumor antigens to APCS (31, 32, 34, 43). While these nanovaccines are effective, their therapeutic benefit can be weakened when tumor cells express low levels of MHC I, which impairs antigen presentation and T cell recognition. The other cancer immunotherapy methods also have this problem.

To overcome the downregulation of MHC I, Several different approaches (5, 8–10, 44), such as treatment with cytokines (e.g., IFN-γ) (45), histone deacetylase inhibitors (HDACis) (46, 47), DNA methyltransferase inhibitors (DNMTis) (48), TKIs (8, 20, 49), and IFN-γ/STAT1 activators (6, 8, 17, 50) etc., have been investigated by different groups to increase the expression of MHC I (5, 8, 16, 44). These different approaches each have advantages and disadvantages (5, 8–10, 16). Herein, in order to increase the opportunities to be allied in clinical, a panel of clinically approved drugs, including TKIs like Sorafenib and Sunitinib, were screened for their ability to upregulate MHC I on tumor cells. And Sorafenib and Sunitinib showed excellent in elevating MHC I expressions on tumor cells through activating IFN-γ/STAT1 pathway, a key pathway in antigen presentation that can upregulate the expressions of MHC I (17, 20). In our previous studies, we have reported a type of cancer nanovaccines loaded with whole tumor antigens (32, 34). The therapeutic efficacy can be further improved by elevating MHC I expressions on tumor cells. Nanovaccines function through activating tumor-specific T cells that recognize tumor cells through antigen-MHC I complex, and Sunitinib/Sorafenib can increase the amount of MHC I to improve the efficacy of cancer vaccines. Therefore, combining Sorafenib or Sunitinib with cancer vaccines potentially can improve the therapeutic efficacy of cancer vaccines.

Though Sorafenib and Sunitinib can efficiently elevate the expressions of MHC I in tumor cells, to maximize the efficacy of Sorafenib and Sunitinib, delivering them targetly to tumor tissues is needed and how to deliver them more efficiently to tumor tissues is a problem. To address this problem, we designed the redox-sensitive nanovesicles, SUN-KD10 and SOR-KD10, to deliver these therapeutic agents directly to tumor sites. These nanovesicles, composed of a PEG-P(TMC-DTC)-KD10 copolymer through self-assembly and disulfide crosslinking, are characterized by high drug loading capacity, small particle size, excellent dispersion, stability, and responsiveness to reductive conditions. They utilize the enhanced permeability and retention EPR effect for passive targeting of tumors and promote the upregulation of MHC I molecules on tumor cell surfaces. By encapsulating drugs like Sunitinib and Sorafenib in these nanovesicles, we minimize off-target effects and toxicity, while benefiting from extended circulation time provided by PEG’s protective properties.

This study demonstrated that combining nanovaccines (loading whole tumor antigens) with SUN-KD10 or SOR-KD10 elicited a synergistic antitumor effect in both melanoma and triple-negative breast cancer models, representing immunologically “hot” and “cold” tumors. The tumor growth and survival time of mice treated with combination therapies were both improved, comparing with administered alone. Flow cytometry confirmed that this combination significantly increased tumor-specific CD8^+^ T cell activation and infiltration, indicating enhanced immune-mediated tumor clearance. Mechanistically, our findings support a dual-action model: nanovesicle-encapsulated TKIs restore MHC I expression (immune sensitization), while tumor antigen-loaded nanovaccines activate antigen-specific T cells (immune activation). This synergistic approach addresses a problem in current immunotherapies, such as checkpoint inhibitors, which often fail in MHC I-deficient tumors. From a translational perspective, this platform uses FDA-approved drugs with well-characterized safety profiles, reducing the barriers to clinical development. Its modular design also permits adaptation to other drug–vaccine combinations or tumor types, offering broad applicability.

In conclusion, this study presents a rational strategy to overcome immune evasion by restoring antigen presentation and boosting tumor-specific immune responses. The combination of redox-sensitive TKI-loaded nanovesicles and whole-tumor-antigen nanovaccines holds significant promise for improving cancer vaccine efficacy and advancing immunotherapy outcomes.

## Data Availability

The original contributions presented in the study are included in the article/**Supplementary Material**. Further inquiries can be directed to the corresponding author.
