# Supplementary material for: Elevating MHC I expression on tumor cells by nanovesicles loading tyrosine kinase inhibitors can improve the efficacy of cancer vaccines
Source: Front Immunol. 2025 Sep 11;16:1653533. doi: 10.3389/fimmu.2025.1653533 (PMC12461261; doi:10.3389/fimmu.2025.1653533)
Supplement: Supplementary file 1 [file DataSheet1.pdf]

Supporting Information

Elevating MHC I expression on tumor cells by nanovesicles loading tyrosine kinase inhibitors can improve the efficacy of cancer vaccines

Huimin Xie [1,2,3,4#], Lin Ma[1,2,3,4,5#,] Xiaoli He[2], Songsong Zhao[6], Jin Wang[1,2,3,4,5,7], Ao Zhu[2,3,4,5,7], Changming Liu[2], Olga Piskareva[8], Chao Deng[6], Fenghua Meng[6], Mi Liu*[1,2,3,4,5,7]

[1] College of Pharmaceutical Sciences, Soochow University, Suzhou, Jiangsu, 215123, People's Republic of China.

[2] Kunshan Hospital of Traditional Chinese Medicine, Kunshan, Jiangsu, 215300, People's Republic of China.

[3] Institute of Minimally Invasive Thoracic Cancer Therapy and Translational Research, Soochow University, Suzhou, Jiangsu, 215123, People's Republic of China.

[4] Jiangsu Province Engineering Research Center of Precision Diagnostics and Therapeutics Development, Soochow University, Suzhou 215123, China.

[5] Suzhou Ersheng Biopharmaceutical Co., Ltd, Suzhou, 215123, People's Republic of China.

[6] Biomedical Polymers Laboratory, College of Chemistry Chemical Engineering and Materials Science Soochow University, Suzhou 215123, P. R. China.

[7] Wuxi Boston Biopharmaceutical Co., Ltd., Wuxi, 214125, People's Republic of China.

[8] Department of Anatomy and Regenerative Medicine, Tissue Engineering Research Group, RCSI University of Medicine and Health Sciences, Dublin, Ireland.

*Corresponding author: Mi Liu

Email: mi.liu@suda.edu.cn

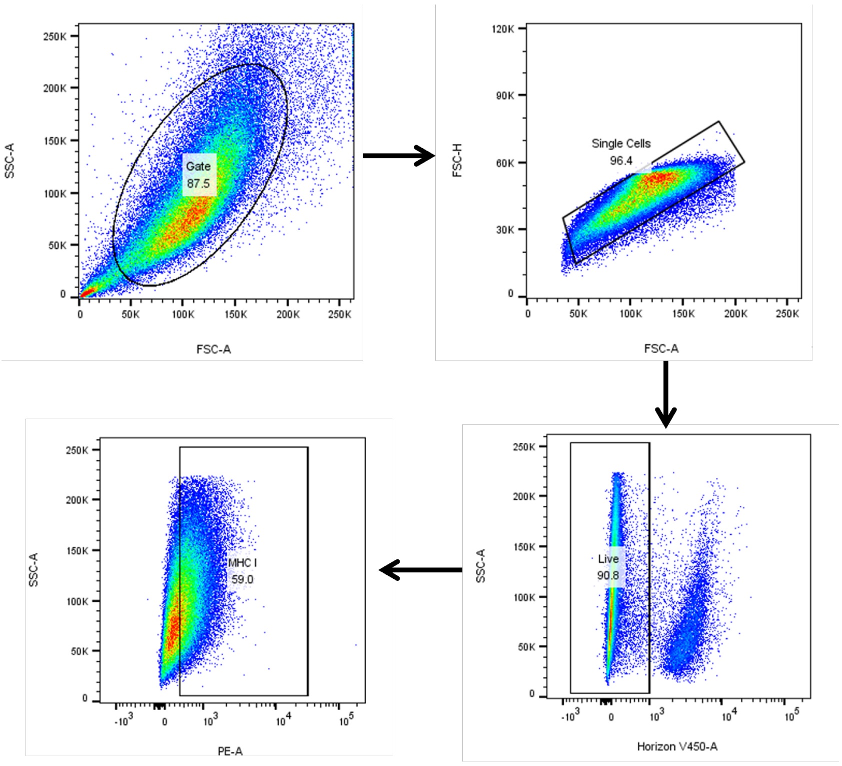

Figure S1. Flow cytometry gating strategy used to analyze MHC I expression on the surface of B16F10 and 4T1.

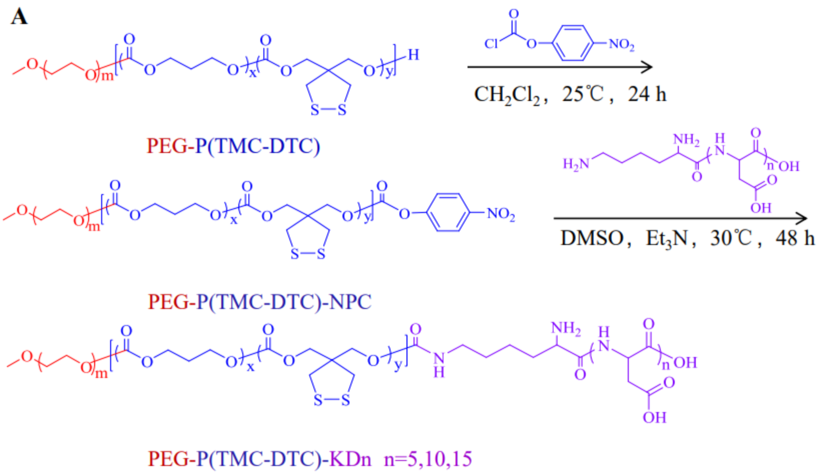

Figure S2. Synthesis route of PEG-P (TMC-DTC)-KD10.

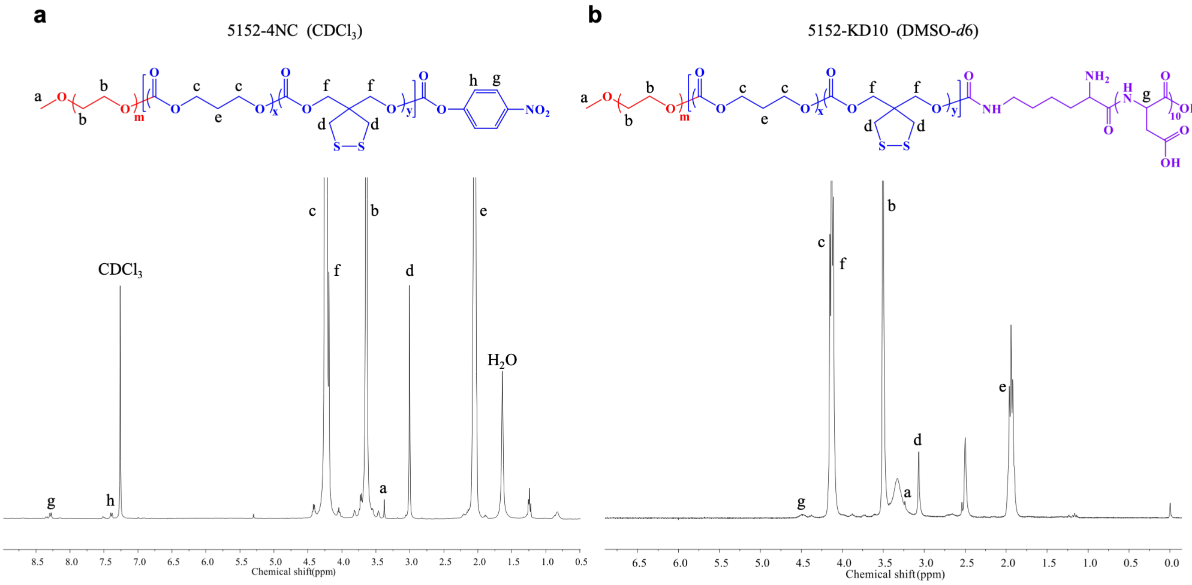

Figure S3. NMR results of PEG-P(TMC-DTC) and PEG-P (TMC-DTC)-KD10.

Table S1. Characterization of Drug-KD_{10} in HEPES buffer.

Drug	KD_{10}	DLC (theory) (wt.%)	DLC (calc.) [a] (wt.%)	DLE[a] (%)	Drug Loading (ug/mL)	Size (nm) [a]	PDI[a]
Sunitinib	2mg	10%	6.57%	63.25%	140.56	45.17±0.86	0.187
		20%	6.37%	27.22%	136.10	38.95±5.57	0.210
	4mg	10%	3.25%	30.19%	134.16	25.97±1.13	0.110
		20%	3.40%	28.16%	140.78	36.73±1.23	0.235
Sorafenib	2mg	10%	3.12%	29.05%	64.48	42.27±0.52	0.043
		20%	5.90%	25.09%	125.47	46.95±1.36	0.169
	4mg	10%	4.26%	40.08%	178.12	37.93±0.35	0.101
		20%	4.73%	39.71%	198.54	40.11±0.16	0.120

a Determined by UV-vis (Sunitinib: 441nm, Palbociclib: 365nm).
b Determined with a Zetasizer Nano ZS instrument (Malvern) equipped with a dynamic light scattering (DLS, 10 mW He-Ne laser, 633 nm wavelength) in Hepes buffer (pH 7.4, 5 mM) at 25 ° C. Nanoparticle concentration was 0.1 mg/mL.

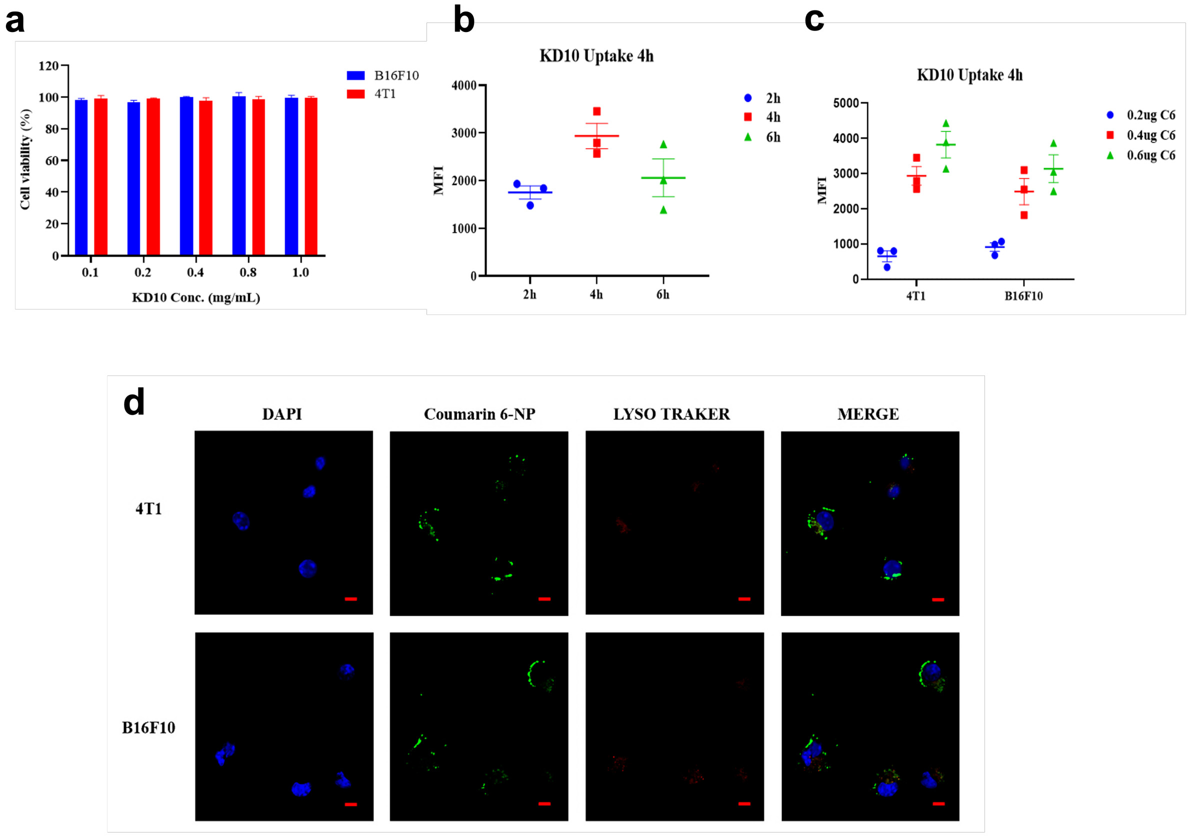

Figure S4. Evaluation of nanovesicle cytotoxicity and endocytosis in 4T1 or B16F10 cells. **a**, Cytotoxicity of blank nanovesicles (KD10) assessed by MTT assay following co-incubation with B16F10 or 4T1 cells. **b,** Flow cytometry analysis of Co6-KD10 internalization in 4T1 cells 2, 4, and 6 hours of co-incubation. **c,** Flow cytometry analysis of Co6-KD10 internalization in B16F10 cells after 4 hours of co-incubation. **d,** Confocal microscopy analysis of Co6-KD10 uptake and endosomal escape in 4T1 or B16F10 cells. Blue: Nuclei (DAPI staining), green: Nanovesicles (Coumarin-6 staining), Red: Lysosomes (LYSO TRAKER staining). Scale bar: 10 μm.

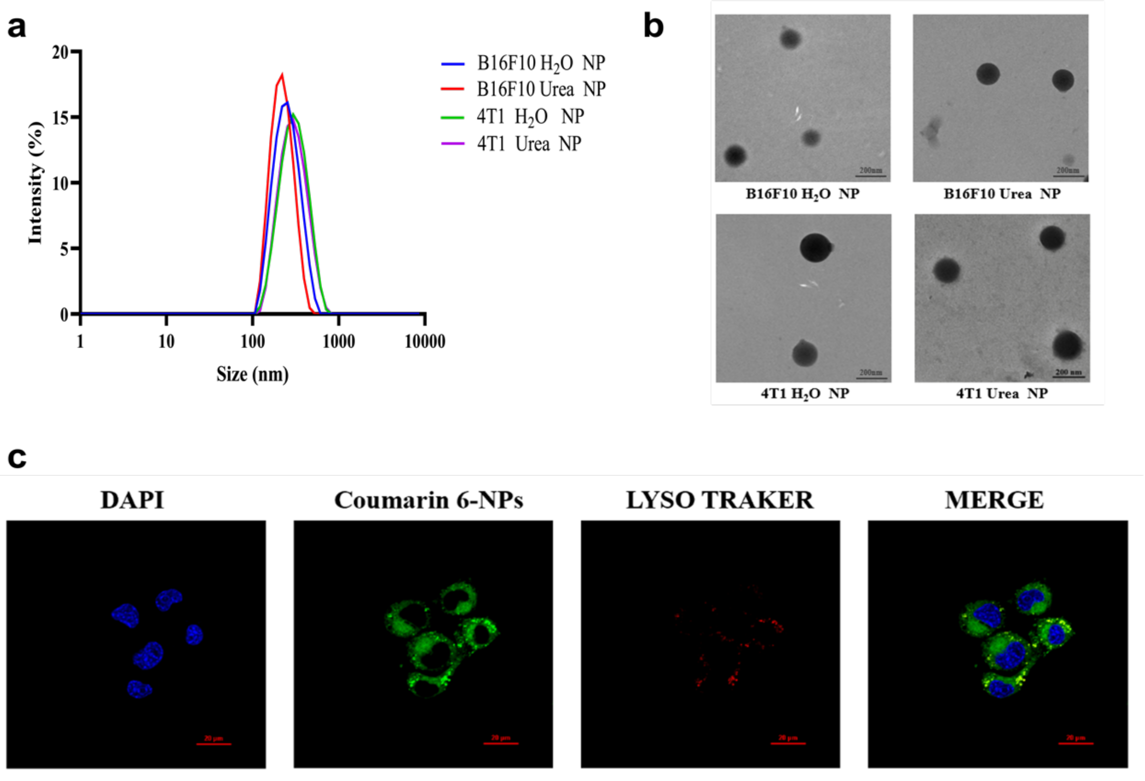

Figure S5. Nanovaccines characterization. a, Particle size distribution of H_2O NP and urea NP as determined by dynamic light scattering (DLS). **b,** Morphological features of H_2O NP and urea NP visualized by **c,** Confocal microscopy analysis of B16F10 cells showing uptake of Coumarin-6 labeled NPs and their subsequent endosomal escape. Blue: nuclei Nuclei were stained with (DAPI staining), green: drug-loaded nanovaccines (were stained with Coumarin-6 staining), Red: Lysosomes were stained with (LYSO TRAKER staining). Scale bar: 20 µm.

Table S2. Characterization of whole-tumour antigen nanovaccines.

	Size (nm)	Zeta (mV)	PDI	Loading capacity (mg/mg PLGA)
B16F10 H_2O NP	239.3 ± 5.8	-21.9 ± 0.9	0.098	0.125
B16F10 Urea NP	234.3 ± 6.3	-18.9 ± 0.7	0.208	0.120
4T1 H_2O NP	271.3 ± 2.3	-15.9 ± 0.9	0.113	0.129
4T1 Urea NP	266.1 ± 6.1	-17.6 ± 0.4	0.261	0.124

Table S3. Dosing regimen and concentrations of combined drugs in animal experiments

	Dose	Route of administration
Sunitinib	20 mg/kg/d	o.p.
Sorafenib	20 mg/kg/d	o.p.

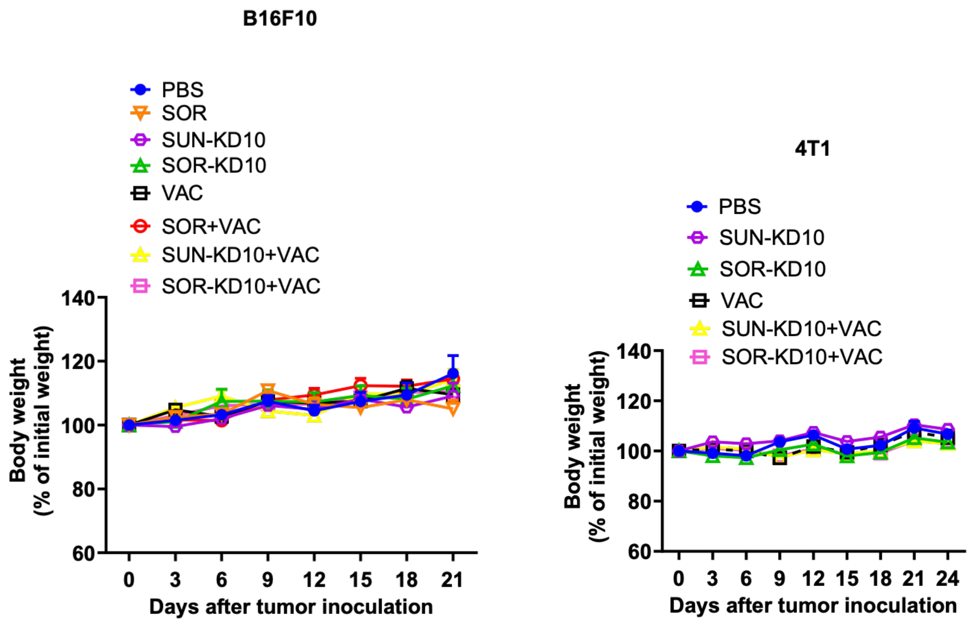

Figure S6. Body weight changes in mice from each treatment group in melanoma and TNBC mouse model (n=8).

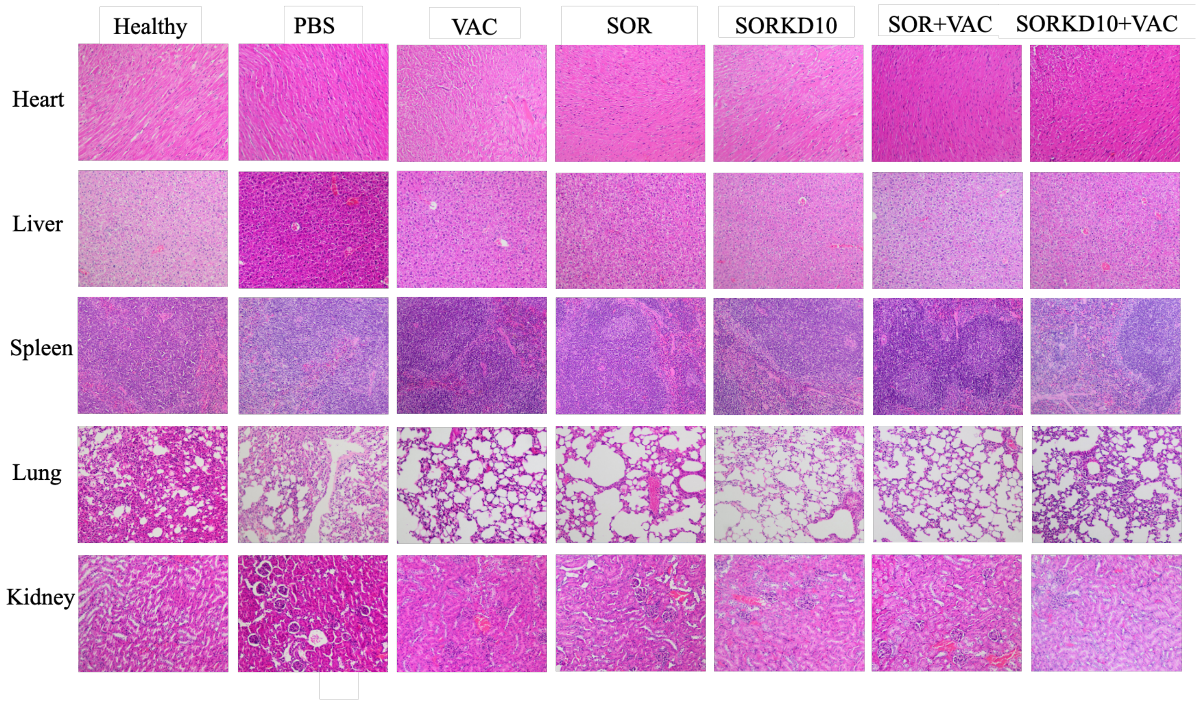

Figure S7. Assessment of potential toxicity to major organs using H&E staining.

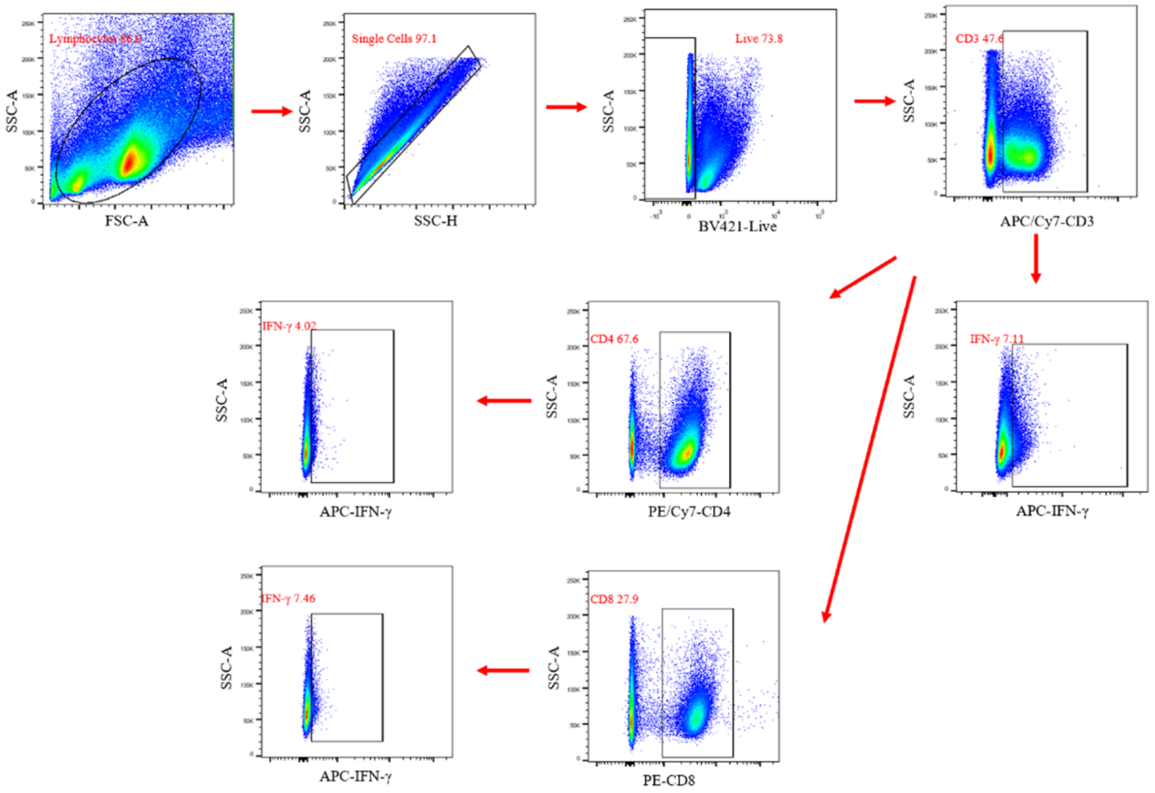

Figure S8. Gating strategy diagram for flow cytometry analysis of INF-γ^{+} CD3/CD4/CD8 T cells in the spleen and lymph node of tumor-bearing mice post-treatment.
